# Human APECED; a Sick Thymus Syndrome?

**DOI:** 10.3389/fimmu.2013.00313

**Published:** 2013-10-07

**Authors:** T. Petteri Arstila, Hanna Jarva

**Affiliations:** ^1^Department of Bacteriology and Immunology, Immunobiology Research Program, Haartman Institute, University of Helsinki, Helsinki, Finland; ^2^HUSLAB, Helsinki University Hospital Laboratory, Helsinki, Finland

**Keywords:** APECED, AIRE, T cells, autoimmunity, thymus

## Abstract

Loss-of-function mutations in the Autoimmune Regulator (AIRE) gene cause a rare inherited form of autoimmune disease, autoimmune polyendocrinopathy-candidiasis-ectodermal dystrophy, also known as autoimmune polyglandular syndrome type 1. The patients suffer from multiple endocrine deficiencies, the most common manifestations being hypoparathyroidism, Addison’s disease, hypogonadism, and secondary amenorrhea, usually accompanied by typical autoantibodies against the target tissues. Chronic mucocutaneous candidiasis is also a prominent part of the disease. The highest expression of AIRE is found in medullary thymic epithelial cells (mTECs). Murine studies suggest that it promotes ectopic transcription of self antigens in mTECs and is thus important for negative selection. However, failed negative selection alone is not enough to explain key findings in human patients, necessitating the search for alternative or additional pathogenetic mechanisms. A striking feature of the human AIRE-deficient phenotype is that all patients develop high titers of neutralizing autoantibodies against type I interferons, which have been shown to downregulate the expression of interferon-controlled genes. These autoantibodies often precede clinical symptoms and other autoantibodies, suggesting that they are a reflection of the pathogenetic process. Other cytokines are targeted as well, notably those produced by Th17 cells; these autoantibodies have been linked to the defect in anti-candida defenses. A defect in regulatory T cells has also been reported in several studies and seems to affect already the recent thymic emigrant population. Taken together, these findings in human patients point to a widespread disruption of T cell development and regulation, which is likely to have its origins in an abnormal thymic milieu. The absence of functional AIRE in peripheral lymphoid tissues may also contribute to the pathogenesis of the disease.

## Introduction

Monogenic diseases, although rare, provide a unique possibility to obtain information in the human system on the significance and function of the molecules affected by the mutations. Autoimmune polyendocrinopathy-candidiasis-ectodermal dystrophy (APECED), also known as autoimmune polyendocrine syndrome type 1 (APS-1), is one such natural knockout phenotype, and has provided important information on T cell selection and pathogenesis of organ-specific autoimmunity (1, 2). It is a recessively inherited human autoimmune disease, caused by loss-of-function mutations in the Autoimmune Regulator (AIRE) gene (3, 4). It is enriched in certain populations, most notably the Finns (prevalence 1:25 000), Sardinians (1:14 000), and Iranian Jews (1:9 000) (5). The pathognomonic triad consists of chronic candidiasis, hypoparathyroidism, and Addison’s disease, with several other endocrine and non-endocrine manifestations affecting a smaller fraction of the patients.

Since the discovery of the underlying genetic defect in 1997, the pathogenesis of this rare polyendocrine syndrome has been the focus of considerable interest. In particular, studies in Aire-deficient mice have shown that AIRE plays an important role in T cell development and negative selection in the thymus, thus elucidating general pathways of thymic development (2). However, the murine phenotype differs in several key points from the human disease, not least in the absence of all the defining triad components mentioned above (6). It is clear that to understand how AIRE works in the human immune system, the human disease mechanisms have to be studied on their own terms, and not only as an extension of the murine phenotype. Such an approach has already proved successful, for example by revealing the role of anti-cytokine antibodies in causing the increased susceptibility to *Candida* infections (7). Here, we review the main features of human APECED, both clinical and immunological. Although several important questions remain open, we also attempt to provide an explanation of the pathogenetic mechanisms, looked at from the human viewpoint.

## Clinical Features

The classic triad of APECED consists of Addison’s disease, hypoparathyroidism, and chronic mucocutaneous candidiasis (CMC), two of which are required for the diagnosis. The autoimmunity in APECED is organ-specific but with many components. There is no gender bias. Virtually all patients have more than two disease components and up to 10 components have been reported. On the average patients have four components (8). First symptoms occur on average at the age of five (range 0.2–18 years) (9). The most common endocrinopathies, hypoparathyroidism, and Addison’s disease are usually diagnosed at the age of 3–5 years and 11–15 years, respectively. New components appear through life (9).

Chronic mucocutaneous candidiasis is usually the first sign of APECED and in Finnish patients its prevalence is 100%. Candidiasis is the most common component of APECED except in Iranian Jews in whom it is rarely described (10). The severity of the symptoms varies from redness and soreness of the corners of the mouth to inflamed mucosal surfaces in the whole oral cavity. Candidiasis increases the risk of oral carcinoma by causing chronic inflammation (1).

Hypoparathyroidism is the most common autoimmune component in APECED and APECED should be considered in the differential diagnosis of every patient with hypoparathyroidism of unknown cause. Addison’s disease is the second most common autoimmune component and its prevalence was 78% in a large patient series (9). It most often presents with both mineralo- and glucocorticoid deficiency. Gonadal failure is a common component, especially in women. Ovarian insufficiency is actually the third most common autoimmune component, affecting approximately 65% of APECED women and starting in early adulthood (9). It can present with primary amenorrhea with a complete failure of or arrested pubertal development. About half of the cases develop premature menopause (9). Testicular failure has a maximum prevalence of 25% in men and starts usually at an older age (11).

About one third of APECED patients develop hypothyroidism, usually after puberty (9). Thyroid autoantibodies are found commonly but clinical disease is not always present. The prevalence of type I diabetes mellitus among APECED patients varies between populations. In a large Finnish patient series, the prevalence was about 30% (8). Gastrointestinal symptoms, such as chronic diarrhea, constipation, hepatitis, and gastritis are common. Autoimmune gastritis with pernicious anemia is present in approximately 30% of patients by middle age (9). In severe forms, patients develop chronic atrophic gastritis and pernicious anemia (9). About 20% of patients develop autoimmune hepatitis with variable severity (8).

Ectodermal manifestations include enamel hypoplasia of the teeth, alopecia, nail dystrophies, vitiligo, and ocular keratopathy (8, 9). Keratitis occurs in 25% of the patients and can lead to vision loss. Keratitis can be an early and even the first manifestation of the disease. Alopecia and vitiligo affect up to 30–40% of the patients by middle age (5, 12).

## Diagnosis and Autoantibody Findings

The diagnosis of APECED is based on the clinical features, detection of autoantibodies, and genetic analysis. Two of the most common disease components are required for APECED diagnosis. Candidiasis is usually the first symptom, while hypoparathyroidism and Addison’s disease are the most common endocrinopathies. Since the gene test is available, the diagnosis is confirmed with the identification of the mutation. The type of mutation, however, does not predict the disease course. Due to the rarity of APECED, there is often delay of years before the diagnosis is set (13).

An important feature in the diagnostics is the existence of IgG autoantibodies. Their possible pathogenetic role is mostly unknown. Many autoantibodies in APECED are targeted against intracellular enzymes (14). It is possible that the detected autoantibodies are not pathogenetic but, instead, are a marker of the ongoing T cell activity at the target tissue (14). The presence of autoantibodies correlates with the disease components but autoantibodies can also precede the onset of the target organ failure (15). Some autoantibodies are closely associated with the corresponding disease manifestation, while others are found only in a subset of patients with the particular manifestation. For example, autoantibodies against calcium-sensing receptor are found in almost all APECED patients with hypoparathyroidism, whereas anti-NALP5 antibodies, also linked to hypoparathyroidism, are much less common (16, 17). Also, autoantibodies found in APECED patients may be different from those found in patients with an isolated autoimmune disease. A case in point is GAD (glutamic acid decarboxylase), an important autoantigen in type I diabetes but rarely targeted in APECED patients with diabetes as a disease component (18).

Once the initial diagnosis is made, APECED patients must be evaluated regularly and tested for autoantibodies, since new disease components appear through life (19). Suggestions for laboratory testing in the follow-up of APECED patients are presented in Table 1.

**Table 1:** **Suggested tests in suspected APECED and for the follow-up of APECED patients**.

Disease component	Autoantibody	Other tests
APECED	Interferon-α and/or interferon-ω	
Hyperparathyroidism	NALP5	Plasma calcium
	Calsium-sensing receptor	Plasma phosphate
Addison’s disease	21-hydroxylase	Plasma renin
	Adrenocortical antibodies	Plasma ACTH
Diabetes mellitus type I	IA-2	
Hypothyroidism	Thyroid peroxidase	
Gonadal insufficiency	Steroid cell antibodies	FSH, LH, estrogen
Gastritis	Parietal cell antibodies	vitamin B12
Hepatitis	LKM antibodies	ALAT

In diagnosed APECED patients the aim is to screen for new disease components for early diagnosis.

The recently discovered neutralizing antibodies against type I interferons are found in 100% APECED patients (20, 21). Anti-interferon antibodies are present before symptom development at high titers. In addition to APECED, type I interferon autoantibodies have been found only in patients with thymoma, but with lower prevalence and titers (20, 21). Thus, measurement of neutralizing antibodies against the type I interferons α and ω is a sensitive diagnostic test.

Chronic mucocutaneous candidiasis correlates with autoantibodies against Th17 class cytokines, both in APECED patients and CMC patients without APECED (22–24). The autoantibodies are neutralizing and have been found against IL-22 (91% of patients), IL-17F (75%), and IL-17A (41%) (22). Testing for these antibodies is not commonly used in the diagnostic evaluation, since the abovementioned type I interferon antibodies are more prevalent and specific.

NACHT leucine-rich-repeat protein 5 (NALP5) is a protein expressed in the cytoplasm of parathyroid chief cells. Forty-nine percent of APECED patients with hypoparathyroidism are positive for NALP5 antibodies (16). These antibodies are not found in APECED patients without hypoparathyroidism or in non-APECED patients with hypoparathyroidism. NALP5 antibodies thus represent APECED-specific autoantibodies.

Adrenocortical autoantibodies can be detected by indirect immunofluorescence assays. These antibodies recognize 21-hydroxylase, 17-hydroxylase, and side-chain cleavage enzymes, which are involved in the steroid hormone synthesis (11). Ovarian insufficiency correlates with the presence of autoantibodies against side-chain cleavage enzyme (11). Steroid cell autoantibodies can be screened for by indirect immunofluorescence assays with ovarian and testicular tissues. APECED patients with ovarian insufficiency also have elevated FSH and LH levels and low estrogen levels (1).

Fifty percent of APECED patients with hepatic involvement have liver-kidney microsomal (LKM) antibodies (25). The cytochrome target antigen is CYP1A2. Autoantibodies against CYP1A2 are highly specific (100%) but their sensitivity is only 50% (12). Plasma alanine amino transferase (ALAT) are a good marker for developing autoimmune hepatitis in APECED (1). Smooth muscle cell antibodies, which are a marker for autoimmune hepatitis, are not commonly present in APECED-associated hepatitis (25).

In APECED, autoantibodies against IA-2 (tyrosine phosphatase-like protein IA-2) correlate with the development of type I diabetes. However, the sensitivity is low (11). GAD antibodies are relatively common (33%) in APECED patients but their presence does not correlate with diabetes, in contrast to non-APECED patients (11, 18).

Gastrointestinal symptoms, for example constipation and diarrhea are common among APECED patients. Hypocalcemia due to hypoparathyroidism can cause diarrhea. Exocrine pancreatic failure occurs in a few percent of patients and can present with malabsorption and steatorrhea (9). Plasma calcium levels and exocrine pancreatic function should be assayed in APECED patients with diarrhea.

## Genetics of APECED

**Figure 1:**
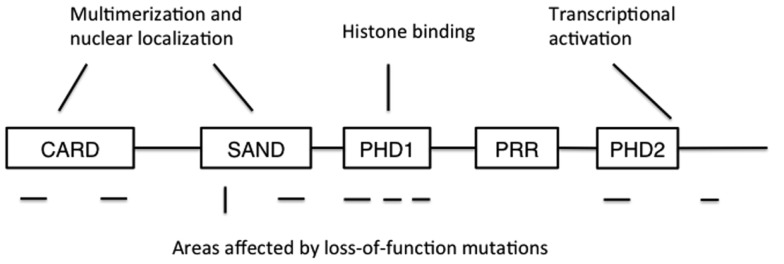
**The structure of AIRE protein**. CARD: caspase recruitment domain; SAND: human Sp100, Aire1, NucP41/P75, and *Drosophila* DEARF1 domain; PHD: plant homeodomain; PRR: proline-rich region. CARD and SAND are associated with AIRE multimerization and nuclear localization, while PHD1 mediates binding to non-methylated histone H3. Mutations disrupting AIRE’s function have been found in all domains except PRR, and also at the extreme C-terminus. Areas where recessive mutations have been found are shown below the schematic structure as horizontal lines, while the location of the dominant mutation is shown as a vertical line.

Autoimmune polyendocrinopathy-candidiasis-ectodermal dystrophy is caused by loss-of-function mutations in the *AIRE* gene. The human *AIRE* gene is found in the q22 region of chromosome 22, and shares a 71% sequence homology with its murine counterpart Aire (3, 4, 26). At full length, AIRE encodes a 58 kDa protein of 545 amino acids, although two other, shorter splice variants of unknown significance have been described (4, 27). AIRE contains a N-terminal caspase-recruitment domain and SAND domain that together regulate AIRE’s multimerization, two plant homeodomains (PHDs), and a proline-rich region (Figure 1) (28, 29). The N-terminus also contains a nuclear localization signal, while C-terminus is important for transcriptional activation. PHDs are zinc fingers involved in protein-protein interaction, and PHD1 has been shown to mediate the binding of AIRE to non-methylated histone H3 (30–34). Together, these sequence and structural features suggest a role in the regulation of gene transcription, but despite some data suggesting otherwise (35), it is unlikely that AIRE is a transcription factor that directly binds DNA.

Today, more than 60 APECED mutations have been identified. The mutations are found throughout the coding region in AIRE and include nonsense mutations causing premature stop codons, frameshifts caused by deletions and missense mutations (36). The most common ones are the mutations R257X (c.769C > T) in exon 6 and a 13-base pair deletion (c.967–979del) in exon 8 (37, 38). APECED is inherited in an autosomal recessive manner and heterozygosity does not cause APECED. However, there is one Italian family where a dominantly inherited mutation in the AIRE gene (G228W) causes APECED (39).

In general, the type of mutation in APECED does not correlate with the clinical features (40). Phenotype can vary even between siblings carrying the same mutation (9). However, the R257X mutation carriers have more commonly candidiasis than patients with other mutations (40). Also, Iranian Jews APECED patients have a unique mutation (Y85C, c.374A > G) and do not develop keratopathy or candidiasis (10).

## AIRE’s Expression and Function

A few years after the discovery of the genetic defect responsible for APECED, experiments with knockout mouse models linked AIRE to the transcription of tissue-restricted antigens (TRAs) in thymus. The highest expression of AIRE is found in medullary thymic epithelial cells (mTECs) (41), which are capable of expressing a diverse set of genes normally restricted to certain tissues (42, 43). This phenomenon has been denoted ectopic transcription and is likely to be important for the deletion of autoreactive thymocytes. In the absence of Aire a subset of thymic TRAs was down-regulated, suggesting that Aire functioned as a regulator of the ectopic transcription of some TRAs in mTECs (44). Another study using a model antigen with Aire-regulated promoter provided evidence of increased development of autoreactive T cells and subsequent autoimmunity when Aire was knocked out, at least in that particular transgenic system (45). Later studies have confirmed but also complicated the link between TRA transcription and Aire (2).

It has been difficult to establish how AIRE facilitates TRA transcription, given the promiscuous nature of its function and the diverse and context-dependent set of genes affected by AIRE’s absence. Several studies have reported that AIRE can bind DNA, suggesting that it might function as a transcription factor (35, 46). Others, however, have challenged the significance of direct DNA binding by AIRE (32, 47, 48). Moreover, the genes regulated by AIRE lack shared promoter elements (47), and it is thus difficult to see how AIRE could by direct DNA binding regulate such a diverse set of genes.

A detailed analysis of AIRE’s molecular partners suggests a less direct role for AIRE in gene transcription. A common feature of AIRE-regulated genes is that they are associated with a stalled RNA polymerase II (RNAPII), which stops full-length transcription (34, 49, 50). AIRE then seems to allow further elongation of the target genes. A recent attempt to formulate a model suggests that AIRE first binds to non-methylated histone H3 (29). It then recruits positive transcription elongation factor b (P-TEFb) to phosphorylate RNAPII, reactivating the stalled polymerase and gene transcription. Although this would allow for a broad range of genes to be activated, a major problem with this model is to explain how AIRE’s control on TRAs is maintained even when the TRAs are expressed as transgenes, outside of their normal epigenetic environment.

A competing explanation is that AIRE is not directly involved in TRA transcription, but rather regulates mTEC maturation and death, thus indirectly affecting also mTEC-expressed TRAs. In support of this possibility are numerous studies showing that Aire-deficiency results in fundamental changes in the mTEC population (51–54). The expression of AIRE is a late event in the lifespan of mTECs (55), and it has been suggested to be a terminal differentiation factor for mTECs. Thus, in the absence of AIRE, the altered developmental pathway of mTECs might lead to a decrease in the TRA-expressing stages, either because of abnormal cell death or diverted or arrested maturation. However, although the mTEC differentiation model is compatible with most reported effects of AIRE-deficiency, the molecular mechanisms by which AIRE regulates mTEC biology are largely unknown.

The strengths and weaknesses of scenarios involving regulation of gene transcription versus regulation of mTEC homeostasis have been more fully summarized in a recent review (47). At the moment the available data do not provide unequivocal support to either of the two main models, and it should also be noted that practically all the data come from studies on the murine system or cell lines. Nevertheless, we would argue that the human phenotype is difficult to reconcile with models invoking regulation of TRA transcription as the main function of AIRE. We discuss these aspects in more detail below, in the chapter on the pathogenesis of APECED.

A further complication is AIRE’s expression in peripheral tissues, a phenomenon of largely unknown significance (56). Early studies showed AIRE mRNA expression in a wide range of tissues, but not all of these studies were confirmed on protein level. In the periphery AIRE is expressed at significantly lower levels than in the mTECs, making reliable detection with mostly polyclonal antibodies difficult, and some studies have questioned the presence of any extrathymic AIRE (57). Nevertheless, a wide agreement exists for the expression of AIRE in lymphoid tissues, with perhaps dendritic cells as the main AIRE+ population (58, 59). The peripheral AIRE+ cells can also express TRAs, although the genes are only partly overlapping with the thymic set (60).

## Immunological Abnormalities

The most obvious immunopathological finding in APECED patients is the diverse set of autoantibodies, mostly against tissues affected by the disease. The autoantibodies are often directed against enzymes involved in hormone synthesis, but since these enzymes are intracellular, the organ-specific autoantibodies rarely have pathogenetic significance. Of special interest are the neutralizing autoantibodies to cytokines, since they are likely to have pathogenetic effects. These autoantibodies are found in practically all APECED patients, while extremely rare in healthy people, and can block interferon-induced gene expression both *in vivo* and *in vitro* (21, 61). The more recently described neutralizing autoantibodies against the Th17 cytokines IL-17A, IL-17F, and IL-22 are linked to the defective antifungal defense and CMC (22, 24).

However, the organ-specific autoimmune manifestations of APECED are generally held to be T cell-mediated, and therefore the T cell compartment in APECED patients has been studied in a number of studies. A recent study showed that APECED patients have a significantly increased frequency of highly differentiated CD8+ effector T cells (62). These cells express CD45RA and lack CCR7, a phenotype similar to that found in some chronic viral infections, and express cytotoxic molecules, such as perforin. The specificity of these cells is not known, but it is likely that at least some of them represent the autoreactive population.

The CD4+ population likewise shows changes, but so far the data are scant and partly contradictory. An increased frequency of CD25 + CD4+ cells, including both regulatory and activated populations, was reported in one series of patients (63), while a later study failed to find differences between patients and controls in the frequency or number of CD4+ activated/memory T cells (64). With the discovery of anti-cytokine antibodies the cells producing Th17 cytokines have also been studied. Despite the presence of anti-IL17 antibodies, IL-17A production is generally normal or even increased, while IL-17F is reduced. An even greater reduction is found in IL-22 responses (22, 65, 66). These defects are closely linked to the chronic candidiasis.

The best-defined T cell defects, confirmed by several studies, are found in the regulatory T cell (Treg) population. The earliest report showed that APECED patients have a decrease of CD25 + CD4+ Tregs, a finding later confirmed in another cohort (64, 67). A more detailed examination was facilitated by the discovery of FOXP3 as the key transcription factor in Tregs, and subsequent development of mAb allowing the identification of FOXP3+ cells. APECED patients have a decreased frequency and number of FOXP3+ Tregs, and in a single-cell analysis the Treg cells express reduced levels of FOXP3 protein (68). Moreover, in an *in vitro* co-culture assay Tregs isolated from APECED patients show defective suppressive function. The most clear-cut Treg abnormalities are found in the CD45RO+ activated subset, the population mostly responsible for the regulatory activity (62, 69). Cells of the innate immune system have also been analyzed, especially those in which AIRE is expressed. The frequency of circulating dendritic cells has been reported to be normal (64, 70), but several studies suggest functional changes, at least in monocyte-derived dendritic cells (58, 71, 72). However, here again the published results have been partly conflicting, with some reporting hyperactivation of dendritic cells, and others defective cytokine production and functional impairment. The expression of pattern-recognition receptors has also been studied and seems normal (70).

An altogether different putative role for AIRE links it with Dectin-1, a receptor of the innate immune system (73). AIRE was reported to form transient complexes with Dectin-1 pathway components and localize with Dectin-1 at the cell membrane. In APECED patients peripheral blood mononuclear cells the production of TNF-α in response to Dectin-1 ligation was reduced. Because Dectin-1 is important in innate recognition of β-glucan and anti-*Candida* responses, these findings offer an alternative mechanism for the defective antifungal defense in APECED patients.

With the exception of the increased susceptibility to *Candida*, the general view emerging from these studies, perhaps not unexpectedly, is one of increased effector activity and decreased regulation. As in all human studies, two major problems complicate the interpretation of these results. First, the analysis is restricted to circulating cells, which are likely to be at best a partial reflection of the local autoimmune process. Secondly, most of the studies have been performed on adult patients with long-established disease, and care is needed to separate primary pathogenetic factors from secondary effects of the disease process. We will discuss pathogenesis below. Nevertheless, it is also important to note that not all secondary processes are irrelevant to the pathogenesis. In most patients, new targets of autoimmunity and new disease manifestations continue to appear later in life, and it is likely that the general immune dysregulation is a contributing factor in this unpredictable progression of the disease.

## Pathogenesis

**Figure 2:**
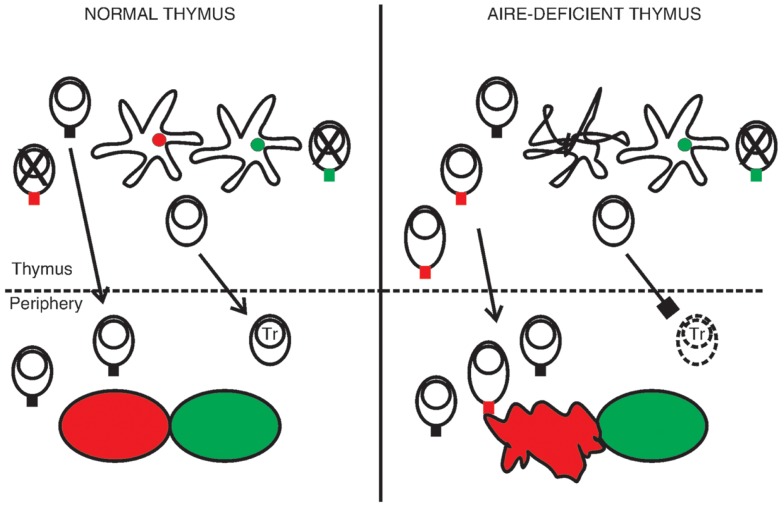
**A hypothesis of the pathogenesis of APECED**. In the normal thymus mTECs express a range of tissue-restricted antigens, shown here as red and green circles within the mTECs. This facilitates the deletion of autoreactive thymocytes, shown as cells with a red or green TCR, so that only T cells reactive to non-self antigens migrate to the periphery. The normal thymus also supports the development of regulatory T cells (Tr). In the absence of functional AIRE the epithelial compartment is disrupted, as indicated by the malformed mTEC, and some of the TRAs are no longer transcribed. Thymocytes specific to these TRAs mature in increased numbers. They may also become activated already in the thymus, shown here as an increase in cytoplasm volume, so that the thymus exports functional effector cells. At the same time the development of Tr cells is defective, leading to insufficient suppression in the periphery. The outcome is organ-specific autoimmunity, at least partly directed by the range of TRAs missing in the AIRE-deficient thymus (here indicated by the red tissue).

Given the expression pattern of AIRE, it is highly likely that the thymus is in a central role in the pathogenesis of APECED. The simplest putative explanation for the disease is based on the link between AIRE and ectopic transcription of TRAs in the thymus. In this model the absence of AIRE-regulated TRAs in the thymus disrupts negative selection and allows the escape of autoreactive T cells to periphery, which leads to organ-specific autoimmunity (Figure 2). Support for this scenario comes from murine studies, and in particular transgenic settings with Aire-regulated model antigens (44, 45, 74, 75).

However, several aspects of both the murine and human AIRE-deficient phenotype are very difficult to reconcile with this simple model. A corollary of TRA-driven autoimmunity is that at least the earliest events should be specific to AIRE-regulated TRAs and the response limited to clones that escaped negative selection. In the murine system some of the reported manifestations involve antigens that are independent of AIRE, suggesting that loss of TRAs is not an obligatory prerequisite of the autoimmunity (76). In humans, perhaps the main problem for the TRA model is to explain the early and universal incidence of anti-interferon antibodies. As noted earlier, these antibodies can precede any clinical symptoms or organ-specific autoantibodies (5), strongly suggesting that their appearance is an important part of the pathogenetic process, or at least reflects it. It is very difficult to see how, without any additional defects, the loss of TRAs in mTECs alone could give rise to this particular phenomenon. Another difficulty arises from the study of thymomas. The disorganized thymoma tissue often lacks AIRE expression, yet continues to support T cell maturation. If the loss of TRAs and subsequent escape of autoreactive T cells would suffice to cause APECED, a substantial fraction of thymoma patients should develop it. Yet the patients rarely show APECED-like manifestations (77, 78). Instead, autoimmunity associated with thymomas is dominated by myasthenia gravis, which conversely is not a manifestation of APECED (9, 79).

An alternative model suggests that the loss of AIRE leads to a more extensive disruption of the thymic microenvironment, creating conditions that favor activation instead of tolerance. Although a primary lymphoid organ, thymus is also capable of developing tertiary lymphoid organization, with germinal centers and induction of adaptive immunity (5). The unique feature of thymus is that, unlike in the secondary lymphoid organs, many of the T cells inhabiting the organ have not yet passed negative selection and are potentially highly autoreactive. The crucial difference when compared with the TRA model is that here the thymus would export preactivated autoreactive T cells, perhaps in high numbers, and not naive, clonally restricted autoreactive precursors. Moreover, since the absence of AIRE is also likely to disrupt the development of natural Tregs (80–82), the defect in immunoregulatory mechanisms contributes to the emerging autoimmunity.

Several observations support the view that a more widespread thymus disturbance than decreased TRA expression alone is responsible for APECED. First, thymoma patients also develop anti-interferon antibodies (83), a feature shared only by these two diseases affecting the thymus, and resected thymoma tissue often shows tertiary lymphoid structures (79, 84). Secondly, in APECED patients even cells expressing markers typical of naive lymphocytes, e.g., CD45RA and CCR7, show clear signs of functional activation, such as the expression of perforin (62). Interestingly, this also applies to CD8+ T cells expressing CD31, a marker of recent thymic emigrants (RTEs). This is consistent with activating events taking place already in the thymus, so that the cells migrate to the periphery preactivated. Similarly, the CD31+ subset among resting Tregs is clearly abnormal, suggesting that the Treg cell defect is also traceable to the thymic development (80). And thirdly, indirect support is provided by the studies showing disruption of thymic medulla in the absence of functional Aire. A dysregulated thymus functioning as an induction site for autoimmune responses would also explain the relatively early onset of the disease.

Nevertheless, the fact that most patients develop similar main components of the disease suggests that the initiation of autoimmunity does show predilection to certain self antigens. It is therefore likely that the range of TRAs missing from the AIRE-deficient thymus, whether primarily due to transcriptional failure or disrupted mTEC development, defines at least to some extent which peripheral organs are targeted. The early pathogenetic events in the thymus would thus be a combination of a general failure to imprint tolerance and a clonally restricted targeting of a subset of potential self antigens.

This thymus-centered view on the pathogenesis of APECED leaves important questions open, including the significance of peripheral AIRE. So far, this is issue is largely unknown, apart from what can be extrapolated from murine studies and the observed changes in the characteristics of dendritic cells and other AIRE+ peripheral cells when AIRE is absent. Likewise, AIRE’s interaction with Dectin-1 partners and its contribution to the antifungal defense remains to be defined.

Moreover, the disease manifestations traditionally held to be caused by factors other than autoimmunity need to be re-examined. The recent data on neutralizing antibodies against Th17 cytokines, and on the importance of these cytokines in anti-Candida defense strongly suggests that the chronic candidiasis is basically an autoimmune phenomenon, too (7). It may also be questioned whether the ectodermal disease components represent developmental dystrophies, as was originally believed. The argument against this view holds that since they are not congenital but develop later, a primary defect is unlikely. However, with the exception of such clearly autoimmune manifestations as alopecia and vitiligo, the pathogenesis of ectodermal components is still unknown.

## AIRE in Other Diseases

Because the effects of AIRE-deficiency are so drastic, many studies have addressed the possibility that heterozygous mutations or genetic variants of AIRE might also predispose to autoimmunity. So far, the results are intriguing but inconclusive and to some extent contradictory. Studies on the first-degree relatives of APECED patients have generally failed to find a link between heterozygous carriage of AIRE mutations and autoimmune diseases (85, 86), although some data suggesting otherwise have been reported (87). A clear limitation in all such studies is the small number of study subjects. Another approach has been to search for AIRE mutations in patients with isolated autoimmune diseases. In most cases heterozygotes have not been found to be enriched among the patients (88, 89), but again there are some conflicting data (90). However, several recent studies suggest that single-nucleotide polymorphisms in the AIRE gene are associated with an increased risk of autoimmunity, including rheumatoid arthritis and vitiligo (91–93). It is therefore probable that more detailed analysis of AIRE will reveal more instances in which genetic variation in AIRE, presumably leading to modulation of its function, affects the predisposition to non-APECED autoimmunity.

## Concluding Remarks

Despite the simple genetics of AIRE, the resulting phenotype is highly complex, and the disease manifestations can vary greatly between patients with identical mutations. The significance of this complexity has sometimes been dismissed by attributing it to secondary effects of a longstanding disease or the genetic heterogeneity of the patients. Although both arguments are relevant, they can also be a too facile way to sidestep important issues. The simple model of reduced TRA expression as the main mechanism of APECED is increasingly untenable, so alternative and additional mechanisms must be considered, and human patients studied to test them. Moreover, although the genetic diversity of the human patients certainly influences and complicates matters, it must be accepted and addressed. The relative simplicity of inbred Aire-deficient animal models is attractive but also potentially deceptive. After all, in the end the results have to be taken back to human patients, when the outbred nature of the subjects is an unavoidable fact.

The existing data indicate that the earliest pathogenetic events leading to APECED take place in the thymus, and it is very likely that a general disturbance of mTEC population is involved. The associated disruption of TRA expression, whatever its exact mechanism, is likely to limit the targets of the resulting autoimmunity, but perhaps not the later appearance of additional, less common disease components. Some of the disease manifestations may also reflect the failure of peripheral tolerance, although the significance of peripheral AIRE expression remains poorly understood. Because the relevant tissues cannot be accessed in APECED patients, many of the open questions can be addressed only indirectly. In particular, innovative organ culture methods to analyze the role of AIRE in the human thymus are likely to provide a means to test the proposed pathogenetic pathways.

## Conflict of Interest Statement

The authors declare that the research was conducted in the absence of any commercial or financial relationships that could be construed as a potential conflict of interest.

## Abbreviations

AIRE, autoimmune regulator; ALAT, alanine amino transferase; APECED, autoimmune polyendocrinopathy-candidiasis-ectodermal dystrophy; APS-1, autoimmune polyendocrine syndrome type 1; CMC, chronic mucocutaneous candidiasis; GAD, glutamic acid decarboxylase; LKM, liver-kidney microsomal; mTEC, medullary thymic epithelial cells; RTE, recent thymic emigrant; TRA, tissue-restricted antigen; Treg, regulatory T cell.
